# JPI-547, a Dual Inhibitor of PARP/Tankyrase, Shows Antitumor Activity Against Pancreatic Cancers with Homologous Recombination Repair Deficiency or Wnt-Addiction

**DOI:** 10.7150/ijbs.113726

**Published:** 2025-08-22

**Authors:** Kyoung-Seok Oh, Ah-Rong Nam, Ju-Hee Bang, Yoojin Jeong, Sea Young Choo, Hyo Jung Kim, Su In Lee, Jae-Min Kim, Jeesun Yoon, Tae-Yong Kim, Do-Youn Oh

**Affiliations:** 1 Cancer Research Institute, Seoul National University College of Medicine, Seoul 03080, Korea.; 2 Integrated Major in Innovative Medical Science, Seoul National University Graduate School, Seoul 03080, Korea.; 3 Department of Internal Medicine, Seoul National University Hospital, Seoul 03080, Korea.

**Keywords:** PARP, PARP inhibitor, homologous recombination deficiency, Tankyrase, RNF43, Wnt/B-catenin.

## Abstract

PARP inhibitors have demonstrated antitumor efficacy in solid tumors, including pancreatic ductal adenocarcinoma (PDAC) characterized by homologous recombination deficiency (HRD). The definition of HRD and other potential biomarkers should be further evaluated using PARP inhibitors. JPI-547 is a novel dual inhibitor targeting PARP1/2 and Tankyrase1/2. Herein, we demonstrate the potent antitumor activity of JPI-547 against BRCA2^-/-^ PDAC cells. JPI-547 outperformed most PARP inhibitors, with a half-maximal inhibitory concentration approximately 10-fold lower than that of olaparib. JPI-547 efficiently trapped PARP1 on the chromatin, disrupted poly-ADP-ribosylation, induced G2/M phase arrest, and triggered apoptosis in PDAC cells. In addition to HRD, we identified Wnt addiction as a predictive factor for JPI-547 activity. PDAC cells reliant on Wnt signaling due to pathogenic RNF43 mutations showed increased susceptibility to JPI-547 without altering homologous recombination repair efficiency. JPI-547 disrupts the Wnt/β-catenin pathway in RNF43-mutated cells and inhibits the oncogenic YAP pathway, highlighting its multifaceted therapeutic potential in PDAC with HRD or Wnt-addiction.

## Introduction

Pancreatic cancer has a poor prognosis, with one of the lowest relative 5-year survival rates of approximately 11% 1. It is the seventh leading cause of cancer-related death worldwide, and over 90% of PC cases are diagnosed as pancreatic ductal adenocarcinoma (PDAC) 2. The current standard of care is surgical resection followed by adjuvant chemotherapy, which is applicable to approximately 20% of patients with PDAC. Approximately 70% of resected cases experience recurrence within a few years 3-6. This scenario underscores the urgent need for novel therapeutic approaches for PDAC.

Recent advancements in precision medicine and technology, including high-throughput next-generation sequencing, have revealed the genetic landscapes and microenvironmental intricacies of PDAC. Predominant mutations in genes such as *KRAS*, *TP53*, and *CDKN2A*, which occur in up to 90% of cases, provided a foundation for molecular subtyping of PDAC 7-9. However, translating these findings into clinical success remains challenging, largely because of the extensive genetic heterogeneity and immunologically "cold" nature of the PDAC tumor microenvironment, which is characterized by stromal desmoplasia 10-12.

The poly(ADP-ribose) polymerase (PARP) inhibitor olaparib has shown clinical efficacy in patients with PDAC harboring deleterious germline BRCA1/2 mutations, securing FDA approval as a maintenance therapy for those sensitive to platinum-based chemotherapy 13. However, the limited prevalence of these mutations confines its use to a small patient subset 14, 15. Thus, predictive biomarkers beyond BRCA1/2 must be identified to achieve clinical consensus on the definition of homologous recombination deficiency (HRD) 16. Moreover, there is an urgent need to develop and assess potent PARP inhibitors (PARPi) with distinct mechanisms of action to broaden the eligible patient population.

Aberrations in the Wnt/β-catenin signaling pathway, which plays a pivotal role in tumorigenesis, metastasis, and tumor stemness, are notably enriched in PDAC 17. These alterations have been directly implicated in PDAC progression and therapeutic resistance, suggesting potential targets for intervention 18-26. In an effort to exploit this vulnerability, multiple classes of Wnt pathway inhibitors—including PORCN inhibitors (e.g., LGK974, RXC004, ETC-153), Frizzled receptor antagonists (e.g., vantictumab), and decoy receptors (e.g., ipafricept)—have been evaluated in early-phase clinical trials or preclinical models of PDAC 27. Among these, PORCN inhibitors block the secretion of all Wnt ligands by inhibiting their palmitoylation, offering a broad blockade of upstream Wnt signaling 28. However, the development of upstream Wnt inhibitors has been hampered by tolerability concerns and challenges in clinical translation, limiting their current application. In addition, some tumors may exhibit ligand-independent β-catenin activation or acquire downstream reactivation mechanisms, which may reduce the long-term efficacy of upstream blockade 29. These limitations underscore the need for alternative approaches that target more distal nodes within the Wnt signaling cascade.

Among the various regulators of canonical Wnt signaling, Tankyrase1/2 (TNKS1/2)—members of the poly(ADP-ribose) polymerase (PARP) superfamily—play a pivotal role by modulating the stability of AXIN1/2—core scaffolding components of the β-catenin destruction complex 30, 31. TNKS catalyzes the PARylation of AXIN proteins, marking them for ubiquitination by RNF146 and subsequent proteasomal degradation 32, 33. This destabilization of AXIN leads to accumulation of cytoplasmic β-catenin and transcriptional activation of Wnt target genes. TNKS also facilitates Wnt signalosome formation by promoting AXIN-LRP6 interaction under Wnt ligand stimulation 34, 35. Thus, TNKS inhibition represents a rational approach to suppress β-catenin signaling, particularly in tumors with constitutive Wnt activation or where upstream blockade may be insufficient. Several TNKS inhibitors (e.g., XAV939, G007-LK, RK-287107) have demonstrated the ability to stabilize AXIN and attenuate Wnt-driven transcriptional programs in preclinical models; however, their application in PDAC and broader clinical development remain limited, highlighting the need to evaluate clinically applicable TNKS inhibitors in PDAC 36. A key genetic context that may sensitize tumors to such an approach is RNF43 inactivation. This ubiquitin E3 ligase, a negative feedback regulator of Wnt signaling, promotes internalization and degradation of Frizzled and LRP5/6 receptors 37. Inactivating mutations in RNF43, found in approximately 7-10% of PDAC cases, disrupt this regulatory mechanism, conferring dependency on autocrine Wnt ligand signaling and enhanced susceptibility to multiple Wnt-targeting agents 38-43. Although RNF43-mutant PDAC is typically ligand-dependent, TNKS inhibition suppresses Wnt signaling at a downstream node, offering a mechanistically distinct approach from upstream Wnt inhibitors by targeting β-catenin signaling downstream of receptor activation.

Building on this mechanistic rationale, JPI-547—a novel dual inhibitor of PARP1/2 and TNKS1/2—was designed to expand the therapeutic utility of PARP inhibition by incorporating Wnt pathway modulation. In early-phase clinical studies, JPI-547 has demonstrated promising safety and antitumor activity in solid tumors with germline or somatic homologous recombination repair (HRR) mutations 44. Given the mechanistic role of TNKS in Wnt signaling, we hypothesized that JPI-547 may exert antitumor activity in Wnt-addicted PDAC in addition to HRD-driven tumors. To test this, we evaluated its efficacy in biomarker-defined preclinical PDAC models harboring HRR deficiency or RNF43 mutations.

## Materials and Methods

### Human cell lines and reagents

Human PDAC cell lines, including Capan-1, Capan-2, AsPC-1, SNU-213, SNU-324, SNU-410, PANC-1, and MIA-PaCa2, were obtained from the Korean Cell Line Bank (Seoul, Korea) and HPAF-II was obtained from the American Type Culture Collection (Manassas, VA, USA). These cell lines were cultured in RPMI 1640 medium (Welgene, Gyeongsan, Korea) supplemented with 10% fetal bovine serum and 10 µg/mL gentamycin at 37°C in 5% CO_2_. We obtained olaparib (AZD2281, #S1060), talazoparib (BMN 673, #S7048), niraparib (MK-4827, #S2741), veliparib (ABT-888, #S1004), and rucaparib (#S4948) from Selleck Chemicals (Houston, TX, USA). JPI-547 was provided by Onconic Therapeutics (Seoul, South Korea). Methyl methane sulfonate (#129925), cycloheximide (#01810), and LiCl (#L7026) were purchased from Sigma-Aldrich (St. Louis, MO, USA). Recombinant human Wnt3a protein (rhWnt3a, #5036-WN-010) was from R&D Systems (Minneapolis, MN, USA).

### Cell viability assay

Cells were plated in 96-well plates and then treated with 50 μL of 3-(4,5-dimethylthiazol-2yl)-2,5-diphenyltetrazolium bromide (MTT) solution (Sigma-Aldrich). After incubation at 37°C for 4 h, the liquid was removed, and MTT formazan crystals were solubilized by adding dimethyl sulfoxide. A Multiskan GO spectrophotometer (Thermo Fisher Scientific, Waltham, MA, USA) was used to measure the absorbance at 540 nm.

### Immunoblotting

Immunoblotting was performed using established protocols 45. Briefly, sodium dodecyl sulfate sample loading buffer was added to the cellular extracts, which were subjected to polyacrylamide gel electrophoresis for protein separation. The separated proteins were transferred onto nitrocellulose membranes. Blocking solution containing 1% nonfat milk and bovine serum albumin in Tris-buffered saline containing Tween was applied to the membranes for 1 h.

The following primary antibodies were used in this study: anti-AMOTL2 (#PA5-78770; Invitrogen Carlsbad, CA, USA); R anti-PAR/pADPr (#4335-MC-100; R&D Systems); anti-GAPDH (#sc-25778) and anti-PARP1 (#sc56197; Santa Cruz Biotechnology, Dallas, TX, USA); anti-phospho histone H2A.X (Ser139) (γ-H2AX) (#05-636) and anti-histone H3 (#06-755; Millipore, Billerica, MA, USA); and anti-β-catenin (#cst-8480), anti-non-phospho (active) β-catenin (Ser33/37/Thr41) (#cst-4270), anti-lamin B1 (#cst-13435), anti-AXIN-2 (#cst-2151), anti-YAP (#cst-14074), anti-CTGF (cst-86641), anti-YAP/TAZ (#cst-8418), anti-TEAD1 (#cst-12292), and anti-phospho YAP (Ser127) (#cst-13008) (Cell Signaling Technology, Danvers, MA, USA).

### Subcellular fractionation

Cellular organelles were isolated from trypsinized cells following the manufacturer's guidelines using a subcellular protein fractionation kit (#78840, Thermo Fisher Scientific).

### Clonogenic assay

Single-cell suspensions were plated in six-well plates and incubated for 9 days. Cells that formed colonies were stained with Coomassie Brilliant Blue. Colonies were quantified using Gel Doc system software (Bio-Rad, Hercules, CA, USA).

### Cell cycle analysis

Cells were collected through trypsinization, fixed in 70% ethanol, and stored at -20 °C for at least 48 h. Following treatment with RNase A, propidium iodide (PI) was added, and cell cycle assessment was conducted using a FACS Calibur flow cytometer (BD Biosciences, Franklin Lakes, NJ, USA).

### Annexin V assay

The Annexin V assay was conducted following the manufacturer's instructions using a BD Pharmingen™ FITC Annexin V Apoptosis Detection Kit I (#556547; BD Biosciences). Harvested cells were labeled with Annexin V and PI to determine the proportion of apoptotic cells using a FACSCalibur flow cytometer (BD Bioscience).

### Animal experiments

Capan-1 and AsPC-1 cells (1 × 10^7^) were resuspended in phosphate-buffered saline and subcutaneously injected into 4-week-old female BALB/c nude mice. The tumor volume was calculated using the formula [(width^2^ × height)/2]. After approximately 2 weeks, when the tumor volume reached approximately 200 mm^3^, the mice were randomly divided into three treatment groups. These groups received either vehicle (10% dimethyl sulfoxide, 10% cremophor EL, and 80% distilled water), olaparib (50 or 100 mg/kg), or JPI-547 (50 or 100 mg/kg) orally once daily for five weeks (5 days on; 2 days off). The study protocol was approved by the Seoul National University Institutional Animal Care and Use Committee (#SNU-240312-5). All animal experiments were conducted at the Seoul National University Institute of Experimental Animals. No blinding was performed during treatment allocation or tumor volume measurement, as all animal experiments were conducted by a single investigator. Sample sizes (n = 6-7 per group) were determined based on prior xenograft studies and were deemed sufficient to detect statistically meaningful differences in tumor growth, while minimizing animal usage in accordance with established ethical standards, including the 3R principle (Replacement, Reduction, and Refinement).

### Immunohistochemistry

The tumor tissues were fixed in 4% paraformaldehyde and embedded in paraffin. Paraffin-embedded blocks were sectioned and subjected to deparaffinization and rehydration. Following antigen retrieval, the slides were stained with anti-Ki67 (#MA5-14520, Invitrogen), anti-γ-H2AX (#NB100-384, Novus Biologicals, Littleton, CO, USA), or anti-non-phospho (active) β-catenin (Ser33/37/Thr41) (#8814, Cell Signaling Technology) and visualized using an OptiView DAB IHC Detection Kit (#760-700; Venetana Medical Systems, Oro Valley, AZ, USA). All procedures were performed according to the manufacturer's instructions.

### Immunofluorescence

The cells were fixed with 4% paraformaldehyde and permeabilized with 0.5% Triton X-100. After blocking with 2% bovine serum albumin in phosphate-buffered saline for 1 h, the cells were incubated overnight with primary antibodies at 4 °C. Primary antibodies included anti-γ-H2AX (#05-636; Millipore) and anti-YAP (#cst-14074; Cell Signaling Technology). The cells were treated with the fluorochrome-conjugated secondary antibodies Alexa Fluor 594 goat anti-rabbit IgG (#A-11012; Invitrogen) and Alexa Fluor 594 goat anti-mouse IgG (#A-11032; Invitrogen). Nuclei were counterstained with 4′,6-diamidino-2-phenylindole (DAPI), and imaging was performed using a STELLARIS 5 confocal microscope (Leica Microsystems, Wetzlar, Germany) following mounting with VECTASHIELD Antifade Mounting Medium (Vector Labs, Burlingame, CA, USA).

### DR-GFP reporter assay

The pDR-GFP (plasmid #26475) and I-SceI (plasmid #60960) constructs were procured from Addgene (Cambridge, MA, USA). Cells were subjected to transient co-transfection using 5 μg of DR-GFP and I-SceI with Lipofectamine 2000 (Thermo Fisher Scientific) in serum-free medium for 6 h, followed by medium supplementation with 10% fetal bovine serum the next day. GFP fluorescence was quantified using a FACSCalibur flow cytometer (BD Biosciences).

### Quantitative reverse transcription polymerase chain reaction

Total RNA was extracted using TRIzol (#10296028; Invitrogen) and reverse-transcribed into cDNA with the ImProm-II™ Reverse Transcription System (#A3800; Promega, Madison, WI, USA), following the manufacturer's instructions. Real-time polymerase chain reaction (PCR) was conducted using a QuantStudio 3 Real-Time PCR Instrument (Applied Biosystems, Foster City, CA, USA) and TOPreal SYBR Green qPCR PreMIX (#RT500M; Enzynomics, Daejeon, Korea). The primer sequences were as follows: cMyc: sense 5′-GTC AAG AGG CGA ACA CAC AAC -3′, anti-sense 5′-TTG GAC GGAC AGG ATG TAT GC-3′; AXIN-2: sense 5′-TAC ACT CCT TAT TGG GCG ATC A-3′, anti-sense 5′-TTG GCT ACT CGT AAA GTT TTG GT-3′; CTGF: sense 5′-CAG CAT GGA CGT TCG TCT G-3′, anti-sense 5′-AAC CAC GGT TTG GTC CTT GG-3′; CYR61: sense 5′-GGT CAA AGT TAC CGG GCA GT-3′, anti-sense 5′-GGA GGC ATC GAA TCC CAG C-3′; VEGFA: sense 5′-TGT CTT GGG TGC ATT GGA G-3′, anti-sense 5′-GAT TCT GCC CTC CTC CTT CTG-3′; β-actin: sense 5′-CCA ACC GCG AGA AGA TGA-3′, anti-sense 5′-CCA GAG GCG TAC AGG GAT AG-3′; IFNA1: sense 5'-GCC TCG CCC TTT GCT TTA CT-3′, anti-sense 5'-CTG TGG GTC TCA GGG AGA TCA-3'; IFNβ1: sense 5′-TTG ACA TCC CTG AGG AGA TTA AGC-3′, anti-sense 5′-TTA GCC AGG AGG TTC TCA ACA ATAG-3′; CXCL10: sense 5′-CCA TTC TGA TTT GCT GCC TTA TC-3′, anti-sense 5′-TAC TAA TGC TGA TGC AGG TAG AG-3′; IFIT1: sense 5′-GCC TAT CGC CAA GAT TTA GAT GA-3′, anti-sense 5′-TTC TGG ATT TAA CCG GAC AGC-3′; ISG15: sense 5′-CGC AGA TCA CCC AGA AGA TCG-3′, anti-sense 5′-TTC GTC GCA TTT GTC CAC CA-3′.

### RNA sequencing

#### RNA Isolation and Library Preparation

Total RNA was isolated using TRIzol, and RNA integrity was assessed using a TapeStation RNA screentape (Agilent, #5067-5576; Agilent Technologies, Santa Clara, CA, USA). Only samples with an RNA integrity number exceeding 7.0 were deemed suitable for RNA library construction. RNA-sequencing libraries were constructed using a TruSeq Stranded mRNA Sample Prep Kit (#RS-122-2101; Illumina, San Diego, CA, USA). These libraries were quantified using KAPA Library Quantification kits, and quality assessment was performed using a TapeStation D1000 ScreenTape (#5067-5582; Agilent Technologies) prior to sequencing on an Illumina NovaSeq platform.

#### Data Processing and Analysis

Raw sequence reads were trimmed using Trimmomatic 0.38 and then aligned to the *Homo sapiens* reference genome (GRCh38) using HISAT v2.1.0. The assembled transcripts and read counts per gene were generated using StringTie v2.1.3b. Differentially expressed genes were identified using DESeq2, with a |fold-change| ≥ 1.5 and raw p < 0.05 considered to indicate significance.

#### Hierarchical Clustering and Functional Analysis

Hierarchical clustering analysis employs complete linkages and Euclidean distance as similarity measures. Functional and pathway analyses were conducted using gProfiler software.

#### Gene Set Enrichment Analysis

Gene Set Enrichment Analysis (GSEA) was performed using GSEA software, version 4.3.2. Gene sets derived from the Gene Ontology, Kyoto Encyclopedia of Genes and Genomes, and HALLMARK databases (version 7.4 of the molecular signature database - mSigDB) were employed. To determine statistical significance, 1,000 permutations were used for p-value calculation.

### RNA interference

For RNA interference, the cells were transfected with either β-catenin-specific or control siRNA (Genolution, Seoul, Korea) using Lipofectamine 2000 for 6 h. The cells were harvested 24 h after initial transfection and reseeded for further experimental procedures. The following small interfering RNA sequences were employed: *CTNNB1*: sense 5′-CCU UUG UCC CGC AAA UCA UUU-3′, anti-sense 5′-AUG AUU UGC GGG ACA AAG GUU-3′; negative control: sense 5'-CCU CGU GCC GUU CCA UCA GGU AGU U-3′, anti-sense 5′-CUA CCU GAU GGA ACG GCA CGA GGU U-3′.

### Immunoprecipitation

To perform immunoprecipitation, cell lysates were initially incubated with a protein A/G agarose bead (Santa Cruz Biotechnology) slurry at a concentration of 25% for 2 h at 4 °C. The lysates were collected via centrifugation and then incubated overnight at 4 °C with either anti-YAP/TAZ (#cst-8418; Cell Signaling Technology) or anti-TEAD1 (#cst-12292; Cell Signaling Technology) antibodies. The beads were added to the mixture and incubated for 4 h, followed by centrifugation. The collected beads were washed five times with lysis buffer, and immunoprecipitated proteins were eluted by boiling in 4x sodium dodecyl sulfate-polyacrylamide gel electrophoresis sample buffer. The resulting samples, along with counter-input samples prepared from the same lysates for subsequent immunoblotting, were subjected to sodium dodecyl sulfate-polyacrylamide gel electrophoresis.

### Enzyme-linked immunosorbent assay

The secretion levels of CCL5 and CXCL10 in conditioned media were quantified using a Human CCL5/RANTES Quantikine ELISA Kit (#DRN00B; R&D Systems) and Human CXCL10/IP-10 Quantikine ELISA Kit (#DIP100; R&D Systems), following the manufacturer's instructions.

### Measurement of serum 2′3′-cGAMP levels

Serum concentrations of 2′3′-cyclic GMP-AMP (2′3′-cGAMP) were quantified using the DetectX® 2′3′-cGAMP Enzyme Immunoassay Kit (#K067-H; Arbor Assays, Ann Arbor, MI, USA) according to the manufacturer's instructions. Serum samples were collected from Capan-1 xenograft-bearing mice at the treatment endpoint. For each treatment group, serum obtained from individual mice was pooled and analyzed in technical triplicate. Absorbance at 450 nm was measured, and 2′3′-cGAMP concentrations were calculated from a standard curve generated using the supplied calibrators.

### Statistical analysis

Statistical analyses were performed using GraphPad Prism version 8.0 (GraphPad Software, La Jolla, CA, USA). For comparisons between two groups, a two-tailed Student's t-test or Mann-Whitney U test was used as appropriate. For comparisons among more than two groups, one-way or two-way ANOVA followed by multiple comparisons tests (Bonferroni or Tukey) was applied, as specified in the figure legends. Mixed-effects models were used for tumor growth curve analysis when appropriate. A p-value < 0.05 was considered statistically significant.

### Data availability

The RNA-Seq data from this study are available through NCBI Gene Expression Omnibus under accession number GSE254997.

## Results

### JPI-547 exhibits potent antitumor activity against homologous recombination-deficient PDAC as a dual inhibitor of PARP and TNKS1/2

**Figure 1:**
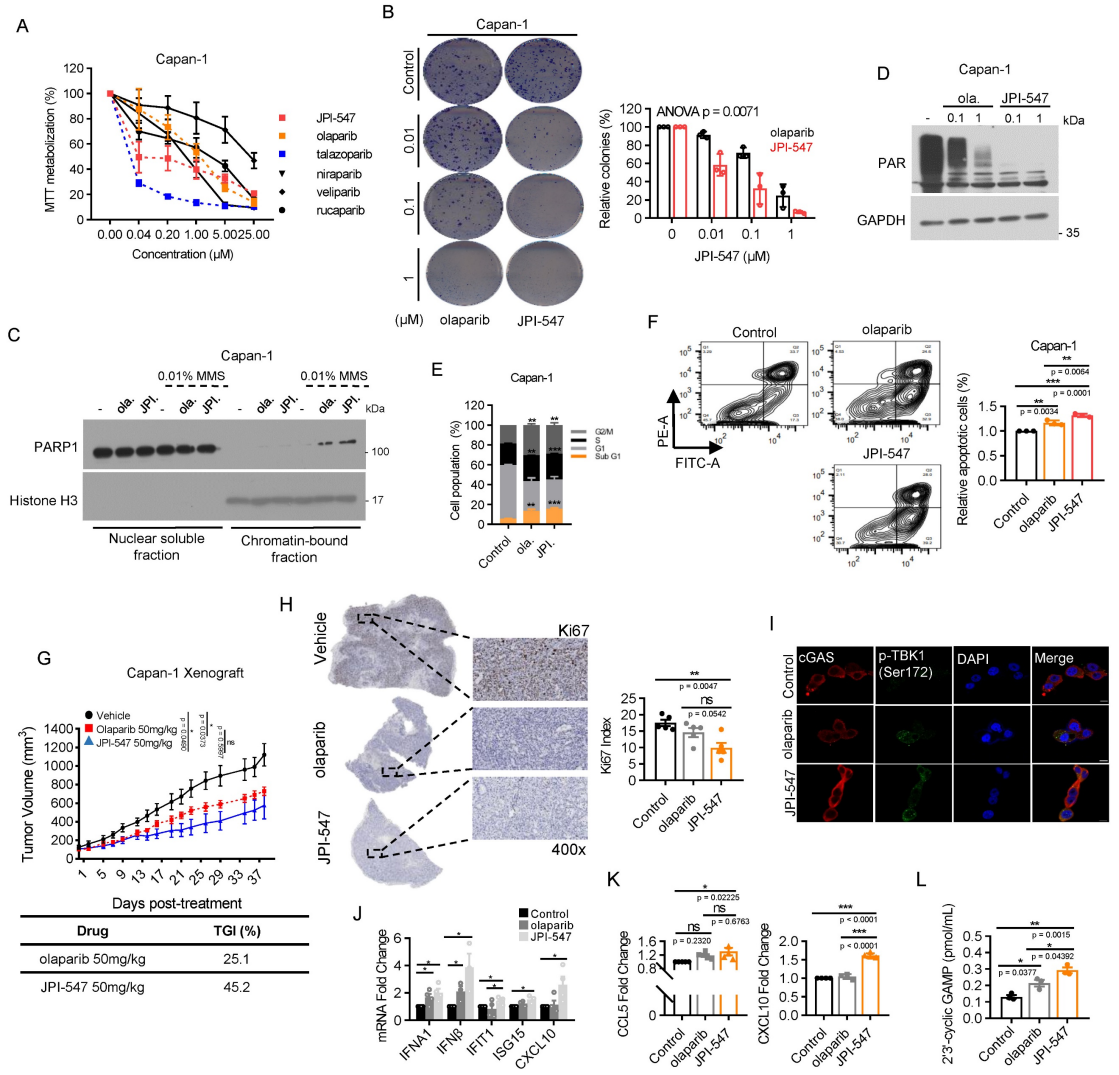
** JPI-547 exhibits potent antitumor activity against homologous recombination-deficient PDAC as a dual inhibitor of PARP and Tankyrase1/2. (A)** Dose-response curves showing cell viability of Capan-1 cells treated with indicated PARP inhibitors. Data represent mean ± SEM (n = 3). **(B)** Clonogenic assays assessing anchorage-independent growth after 9 days of treatment with olaparib or JPI-547. Representative images (left) and quantification of colony numbers. Data represent mean ± SEM (n = 3); p-values by two-way ANOVA. **(C)** Immunoblot analysis of chromatin-bound PARP1 under 0.01% methyl methane sulfonate stress following treatment with 2.5 μM of olaparib or JPI-547. Histone H3 serves as a chromatin loading control. Representative images from duplicate analyses are shown. **(D)** Immunoblot analysis of PAR/pADPr following treatment with indicated concentrations of olaparib or JPI-547 for 72 h. Representative images from two independent analyses are presented. **(E)** Cell cycle analysis following 120 h treatment with 2.5 μM olaparib or JPI-547. Data represent mean ± SD (n = 3); *p < 0.05, **p < 0.001 by two-tailed Student's t-test. **(F)** Upper: Representative flow cytometry images from Annexin V apoptosis assay after 120 h of treatment. Lower: Quantification of apoptotic cells. Data represent mean ± SEM (n = 3); adjusted p-values by one-way ANOVA with Bonferroni's multiple comparisons test. Upper: Tumor growth curves of subcutaneous Capan-1 xenografts treated with vehicle (n = 6), olaparib (n = 7), or JPI-547 (n = 7). Treatment administered orally q.d. for ~5 weeks. Data represent mean ± SEM; adjusted p-values by mixed-effects model with Tukey's multiple comparisons test at the endpoint. Lower: Calculation of TGI rates using the provided formula. TGI = (1 - (mean volume of treated tumors)/(mean volume of control tumors)) × 100. **(H)** Representative images (left) of Ki67 immunohistochemical staining from excised tumors collected at endpoint from the same Capan-1 xenograft cohort shown in **(G)**, treated with vehicle, olaparib, or JPI-547 for 5 weeks. Ki67 index (right) (Ki67-positive tumor cells/total counted tumor cells) presented as the mean ± SD (n = 5); adjusted p-values by one-way ANOVA with Bonferroni's multiple comparisons test. (I) Immunofluorescence analysis of cGAS (red) and p-TBK1 (Ser172) (green) in Capan-1 cells after treatment with 2.5 μM of olaparib or JPI-547 for 72 h. **(J)** qRT-PCR analysis of IFN-I and interferon-stimulated genes after 72 h treatment with 1 μM olaparib or JPI-547. Data represent mean ± SEM (n = 4); adjusted p-values by one-way ANOVA with Bonferroni's multiple comparisons test. **(K)** ELISA quantification of secreted CCL5 and CXCL10 from Capan-1 cells after 72 h treatment. Data represent mean ± SEM (n > 4); adjusted p-values by one-way ANOVA with Bonferroni's multiple comparisons test. **(L)** Serum 2′3′-cGAMP levels collected at endpoint from the same cohort of Capan-1 tumor-bearing mice used in **(G)**, following 5-week oral treatment with vehicle, olaparib (50 mg/kg), or JPI-547 (50 mg/kg). Pooled serum per group. Data represent mean ± SEM (n = 3); adjusted p-values by one-way ANOVA with Bonferroni's multiple comparisons test.

To assess the therapeutic potential of JPI-547 against PDAC characterized by HRD, we compared its antiproliferative effects against various PARPi using the BRCA2-deficient Capan-1 cell line. JPI-547 robustly inhibited the proliferation in Capan-1 cells, exhibiting superior efficacy to most other PARPis, except for talazoparib (Figure 1A). Notably, the half-maximal inhibitory concentration of JPI-547 (0.1895 μM) was approximately 10-fold lower than that of olaparib (1.105 μM), which is the only clinically approved intervention for PDAC (Table 1). In addition, clonogenic assays confirmed the superior effects of JPI-547 over olaparib in suppressing anchorage-independent growth of Capan-1 cells (Figure 1B). At the mechanistic level, JPI-547 displayed an equivalent or slightly enhanced capacity to trap PARP1 in chromatin under conditions of DNA damage induced by the DNA alkylating agent methyl methane sulfonate (Figure 1C). JPI-547 more potently disrupted PARylation than did olaparib, indicating its potent inhibition of PARP catalytic activity (Figure 1D). Cell cycle analyses revealed increased G2/M phase arrest along with an expanded sub-G1 population of Capan-1 cells following olaparib and JPI-547 treatment, suggesting the involvement of apoptotic cell death (Figure 1E). Using Annexin V assays, we confirmed that JPI-547 induced apoptotic cell death more effectively than did olaparib in Capan-1 cells (Figure 1F). To substantiate our *in vitro* findings, we established an *in vivo* xenograft tumor model using Capan-1 cells. Although both olaparib and JPI-547 significantly delayed tumor growth, JPI-547 exhibited a higher tumor growth inhibition rate (45.2%) than that of olaparib (25.1%) (Figure 1G). Immunohistochemistry-based tumor assessment of Ki67 validated that the proliferation of Capan-1 tumors was restrained by either olaparib or JPI-547, with a slightly more pronounced effect observed with JPI-547 (Figure 1H).

Interestingly, RNA-sequencing revealed a distinctive effect of JPI-547 on the transcriptional regulation of genes associated with tumor immunogenicity in HRD cells. GSEA identified gene sets of antigen processing and presentation machinery, and the immunoglobulin-mediated immune response was significantly upregulated with the highest ranking in the enrichment score following JPI-547 treatment compared with olaparib treatment in Capan-1 cells (Figure S1A), suggesting a specific mechanism of immune modulation by JPI-547. Notably, preclinical research has emphasized the immunomodulatory capabilities of PARPi in activating the cytoplasmic DNA recognition cGAS-STING pathway, leading to elevated PD-L1 expression, a type-1 interferon (IFN-I) response, and tumor infiltration by lymphocytes 46-49. These results establish a strong rationale for combining a PARPi with immune checkpoint blockade therapies, propelling the clinical exploration of this strategy in PDAC (POLAR Study; NCT04666740, PARPVAX Study; NCT03404960, and NCT04548752). Significantly, JPI-547 exhibited more potent augmentation of the phosphorylation of STAT1, IRF3, and TBK1, accompanied by increased cytoplasmic cGAS expression, compared with olaparib (Figure S1B, Figure 1I). *In vitro* assays confirmed that JPI-547 promoted the production of IFN-I and IFN-stimulated genes, including IFIT1 and CXCL10 (Figure 1J). Moreover, JPI-547 stimulated extracellular release of CCL5 and CXCL10 in Capan-1 cells (Figure 1K). In contrast, olaparib increased IFN-I production but did not stimulate IFIT1 or CXCL10 expression and failed to induce CCL5 and CXCL10 release. These findings indicate robust activation of the cGAS-STING pathway by JPI-547 compared to that by olaparib. To extend this observation *in vivo*, we measured serum levels of 2′3′-cGAMP—an “immunotransmitter” produced upon cGAS activation—in mice bearing Capan-1 xenografts at the treatment endpoint. JPI-547 treatment led to a significant increase in circulating 2′3′-cGAMP compared to both control and olaparib groups, supporting systemic activation of the cGAS-STING pathway *in vivo* (Figure 1L).

These data collectively highlight the potent antitumor and immunostimulatory effects of JPI-547 in PDAC harboring HRD.

### Wnt-addiction confers vulnerability to JPI-547 in PDAC

**Figure 2:**
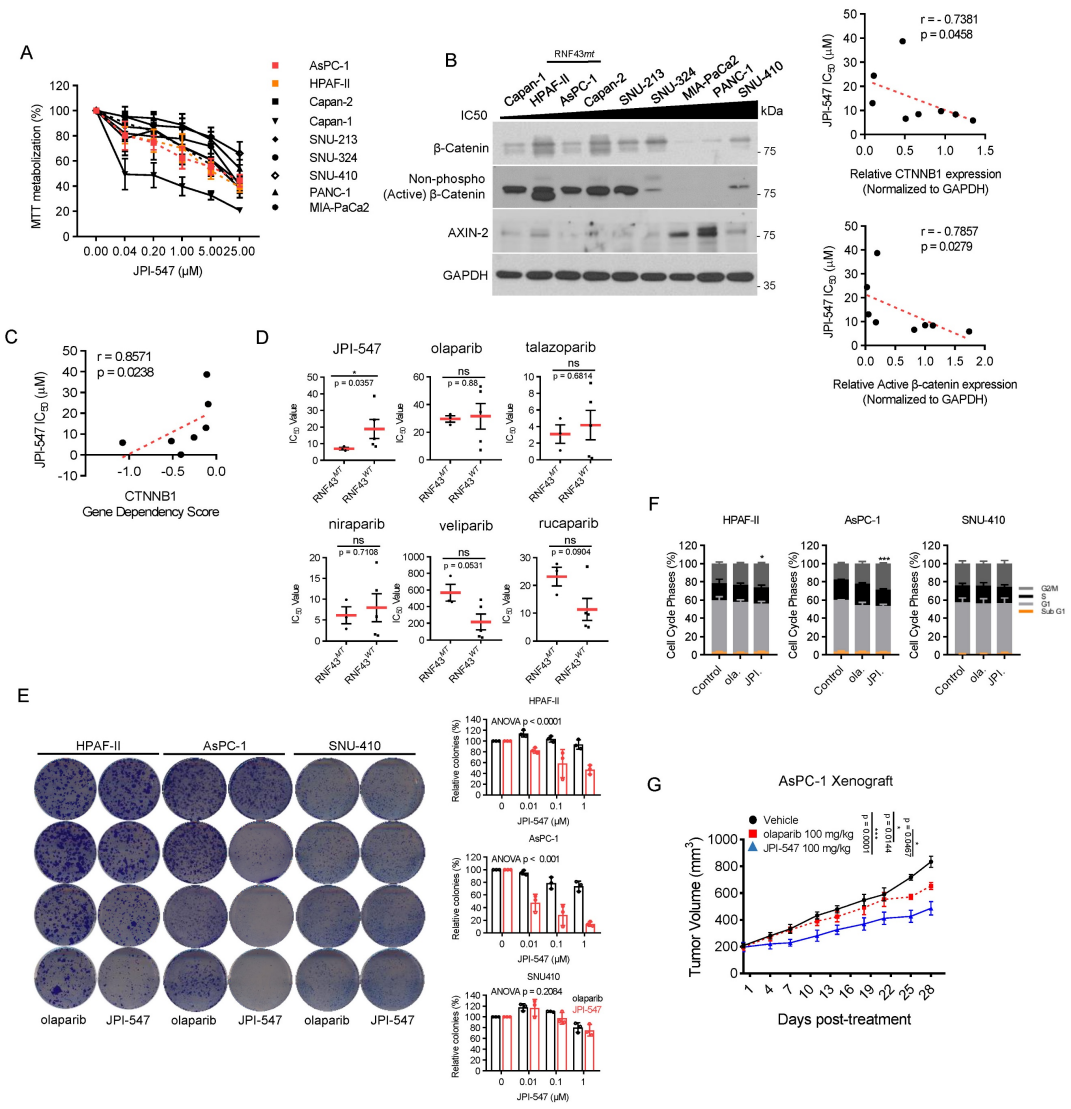
** Wnt-addiction confers vulnerability to JPI-547 in PDAC. (A)** Dose-response curves showing cell viability of human PDAC cell lines treated with JPI-547. Data represent mean ± SEM (n = 3). **(B)** Immunoblot analysis of the basal expression levels of β-catenin or non-phospho (active) β-catenin (Ser33/37/Thr41) in human PDAC cell lines (left), along with their correlation with JPI-547 sensitivity (right). Correlation analysis utilized nonparametric Spearman correlation (two-tailed). β-catenin or non-phospho (active) β-catenin (Ser33/37/Thr41) expression levels were quantified using ImageJ software. **(C)** Correlation between CTNNB1 dependency (DepMap 22Q2 dataset) and JPI-547 sensitivity in PDAC cell lines. SNU-213 excluded due to missing dependency score. Spearman correlation (two-tailed). **(D)** Comparison of IC50 values for JPI-547 between RNF43 wild-type and mutant PDAC cell lines. Data represent log-transformed IC50 values; p-value by two-tailed Mann-Whitney U test. **(E)** Clonogenic assays after 9 days of treatment with 2.5 μM olaparib or JPI-547. Representative images (left) and quantification of colony numbers (right). Data represent mean ± SEM (n = 3); p-values by two-way ANOVA. **(F)** Cell cycle analysis following 120 h of treatment with 2.5 μM olaparib or JPI-547. Data represent mean ± SEM (n = 3); *p < 0.05, ***p < 0.0001 by two-tailed Student's t-test. **(G)** Tumor growth curves of subcutaneous AsPC-1 xenografts treated with vehicle, olaparib, or JPI-547, administered orally q.d. for 4 weeks. Data represent mean ± SEM (n = 6); adjusted p-values by mixed-effects model with Tukey's multiple comparisons test at the endpoint.

Next, we aimed to identify potential biomarkers predictive of the response to JPI-547, extending beyond HRD status. Given that TNKS1/2 is involved in the Wnt/β-catenin pathway, we hypothesized that JPI-547 exerts its inhibitory effects on PDAC cells via a process highly reliant on this oncogenic pathway 32. To test this hypothesis, cell viability analyses were conducted following treatment with JPI-547 in a panel of eight human PDAC cell lines, with Capan-1 included as a positive control (Figure 2A). We found that the mRNA expression levels of *CTNNB1* (encoding β-catenin) and transcriptome profile related to the β-catenin phosphorylation cascade were not significantly associated with the responsiveness of PDAC cells to JPI-547 (Figure S2A, B). In contrast, we observed a strong positive correlation between the protein expression level of either the total or active form of β-catenin (non-phosphorylated on Ser33/37/Thr41) and sensitivity to JPI-547 (Figure 2B). Notably, the *CTNNB1* dependency score, which reflects the essentiality of a specific gene by measuring the lethality of its knockout in a target cell line, also exhibited a significant correlation with sensitivity to JPI-547 (Figure 2C).

Interestingly, the presence of an RNF43 mutation emerged as a determinant of drug sensitivity to JPI-547, distinguishing it from other PARPis (Figure 2D). This includes loss-of-function mutations identified in Capan-2 (p.Arg330fs), HPAF-II (p.Glu174Ter), and AsPC-1 (p.Ser720Ter), all of which are predicted to disrupt RNF43 function and are associated with Wnt pathway addiction. Analysis of The Cancer Genome Atlas dataset revealed genetic alterations in RNF43 in approximately 6% of patients with PDAC, ranking it as the fourth most prevalent alteration among solid tumors (Figure S3). This finding underscores the potential for effective patient enrichment based on genetic signatures. To clarify this observation, we further examined the HPAF-II and AsPC-1 cell lines, which are characterized by loss-of-function mutations in RNF43 (RNF43^mt^). SNU-410, which harbors wild-type RNF43 (RNF43^wt^) and showed the highest resistance to JPI-547 among tested cell lines, was employed as a control. Clonogenic assays revealed that cells carrying RNF43^mt^ showed increased sensitivity to JPI-547 compared to olaparib, which was not observed in RNF43^wt^ cells (Figure 2E). This result was supported by cell cycle analyses, which demonstrated that only JPI-547 induced G2-M phase arrest in RNF43^mt^ PDAC cells (Figure 2F). Additionally, in an immunocompromised mouse xenograft model, JPI-547 suppressed AsPC-1 tumor growth more effectively than did olaparib, underscoring the susceptibility of Wnt-addicted PDAC to JPI-547 (Figure 2G).

### Homologous recombination modulation is not implicated in the susceptibility of Wnt-addicted PDAC cells to JPI-547

**Figure 3:**
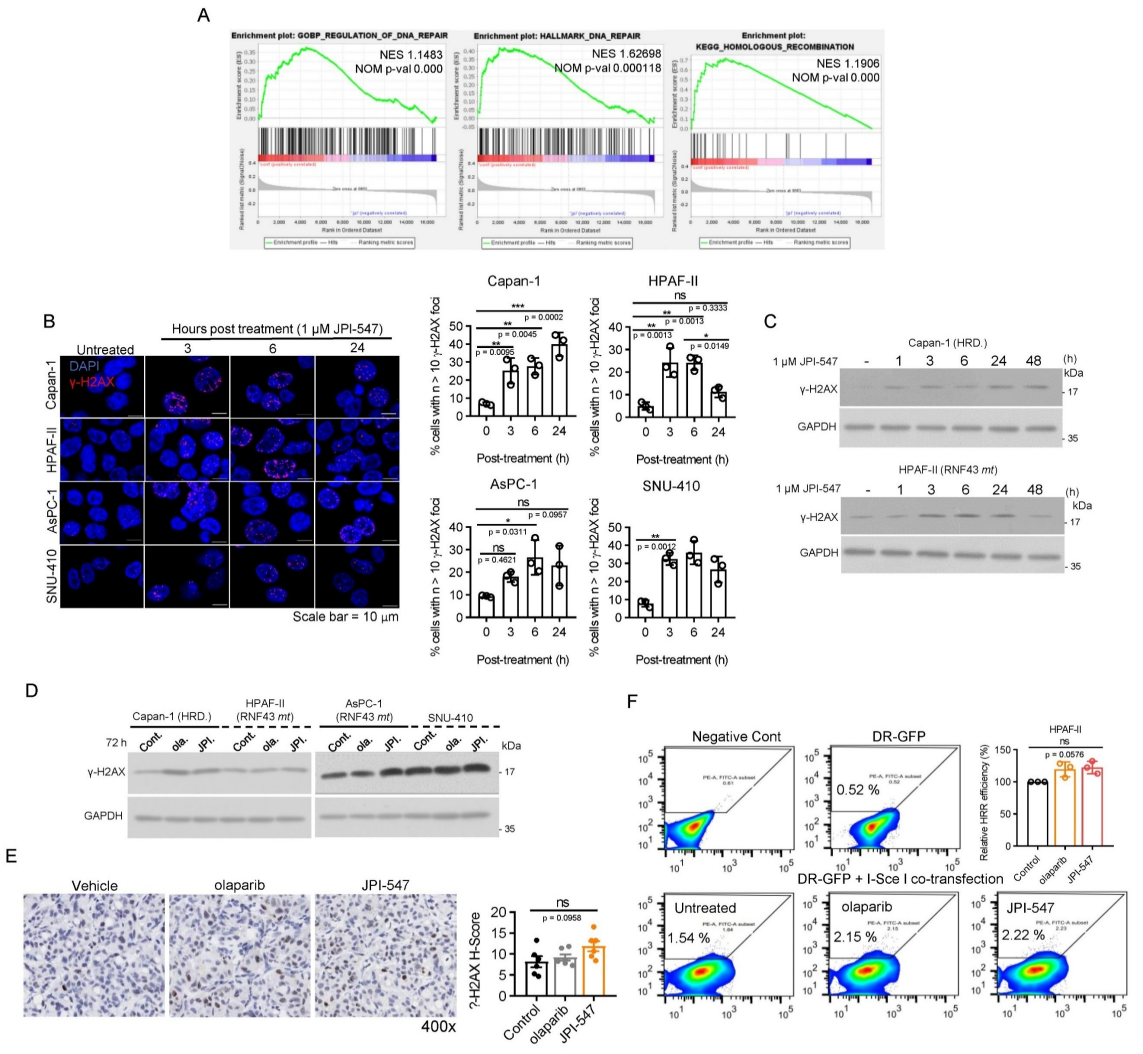
** Homologous recombination modulation is not implicated in the susceptibility of Wnt-addicted PDAC cells to JPI-547. (A)** Gene Set Enrichment Analysis plots showing enrichment of DNA repair gene signatures in control vs. JPI-547-treated HPAF-II cells based on RNA-seq data. Normalized enrichment scores and nominal p-values were calculated using 1,000 permutations. **(B)** Immunofluorescence staining of γ-H2AX (red) in the indicated cell lines after JPI-547 treatment. Bar graphs show the percentage of γ-H2AX foci-positive cells at each time point. Data represent mean ± SEM (n = 3); p-values by one-way ANOVA with Bonferroni's multiple comparisons test. **(C)** Immunoblot analysis of γ-H2AX levels in response to time-dependent JPI-547 treatment in the indicated cell lines. Representative images from two independent experiments are shown. **(D)** Immunoblot analysis of γ-H2AX levels after 120 h of treatment with 2.5 μM of olaparib or JPI-547. Representative images from two independent experiments are shown. **(E)** Representative images of γ-H2AX immunohistochemical staining of AsPC-1 xenograft tumors collected at therapy endpoint following 5-week oral treatment with vehicle, olaparib, or JPI-547 (left). H-score quantification (right) presented as mean ± SEM (n = 6); adjusted p-values by one-way ANOVA with Bonferroni's multiple comparisons test. H-score was calculated as: ((% strong positive cells) × 3) + ((% moderate positive cells) × 2) + ((% weak positive cells) × 1). **(F)** DR-GFP reporter assay measuring homologous recombination repair efficiency in cells co-transfected with I-SceI and pDR-GFP, followed by 6 h treatment with 2.5 µM olaparib or JPI-547. Bar graphs represent percentage of PI⁻/GFP⁺ cells. Data represent mean ± SEM (n = 3); adjusted p-values by one-way ANOVA with Bonferroni's multiple comparisons test.

Previous research has demonstrated that inhibition of the Wnt/β-catenin pathway using a porcupine O-acyltransferase inhibitor can recapitulate a BRCA-like state by disrupting MYBL2-dependent transcription of homologous recombination (HR) and Fanconi anemia genes 50. Consistently, our RNA sequencing analysis revealed a substantial reduction in the expression of MYBL2 (fold-change: -2.64, raw p-val: 1.6603E^-28^, data not shown) and DNA repair gene sets, notably those associated with HR, following JPI-547 treatment in HPAF-II cells (Figure 3A). In contrast, olaparib had no discernible effects on these gene sets (data not shown). These findings suggest that TNKS inhibition may contribute to transcriptional suppression of HR genes. However, functional analyses indicated that this transcriptional suppression does not lead to impaired HRR activity in RNF43^mt^ cells. Immunofluorescence assays showed only transient γ-H2AX foci formation following JPI-547 treatment in RNF43^mt^ and SNU-410, unlike the sustained response observed in HR-deficient Capan-1 cells (Figure 3B). Immunoblot analyses similarly revealed fluctuating γ-H2AX levels that returned to baseline at later time points in cells with RNF43^mt^ following JPI-547 treatment (Figure 3C, D). IHC analysis of AsPC-1 xenografts confirmed that JPI-547 did not significantly alter γ-H2AX expression, corroborating the *in vitro* findings (Figure 3E). Furthermore, DR-GFP assays demonstrated that neither JPI-547 nor olaparib induced an HR-deficient or 'HR-mimicking' state in RNF43^mt^ PDAC cells, as both agents failed to impair HRR efficiency (Figure 3F). Collectively, although JPI-547 treatment was associated with partial transcriptional repression of HR genes, HR modulation does not appear to be a functionally relevant mechanism in the susceptibility of Wnt-addicted PDAC cells to JPI-547.

### Crucial role of Wnt/β-catenin pathway inhibition in JPI-547 cytotoxicity in Wnt-addicted PDAC cells

**Figure 4:**
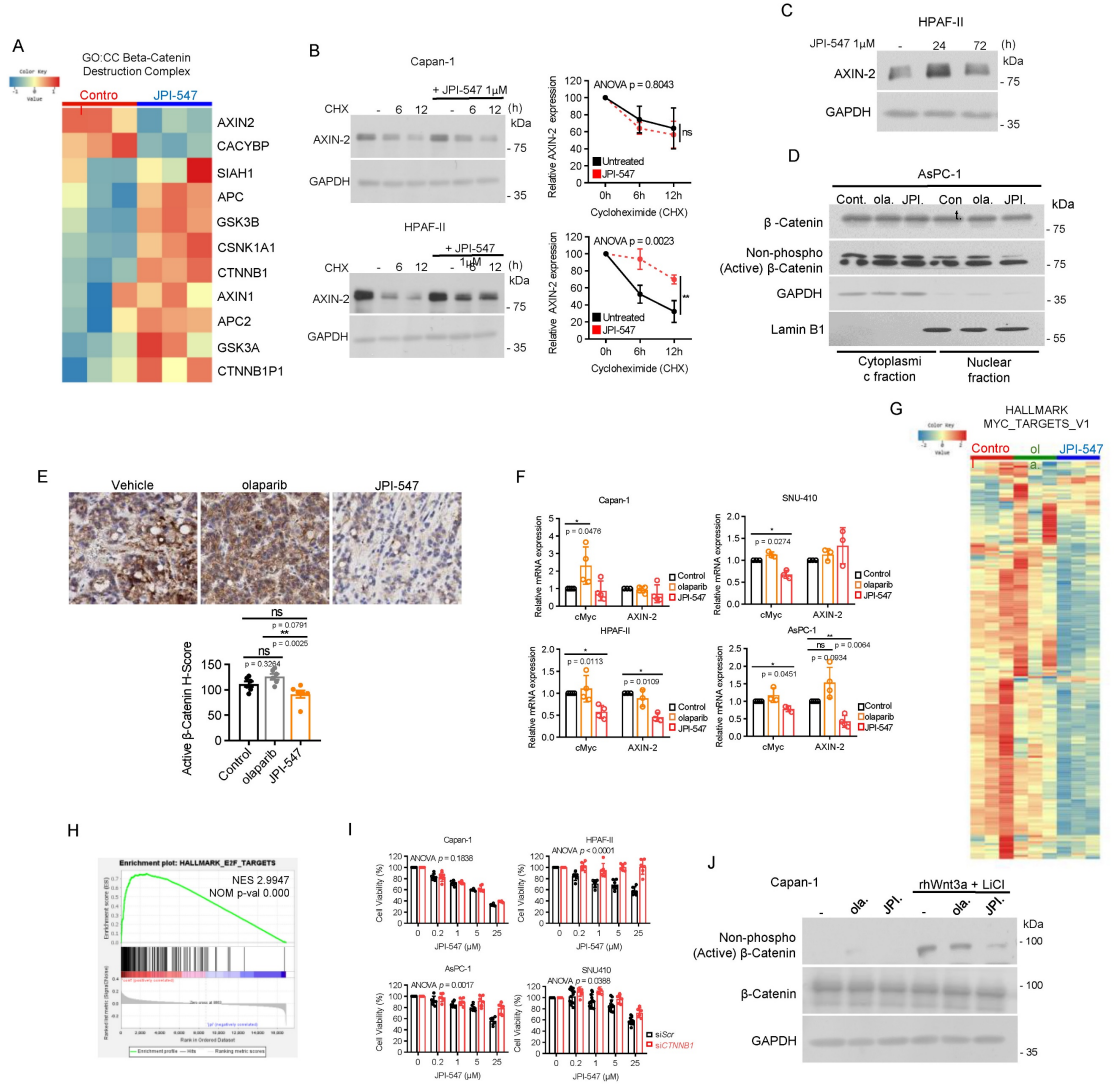
** Crucial role of Wnt/β-catenin pathway inhibition in JPI-547 cytotoxicity in Wnt-addicted PDAC cells. (A)** Heatmap of RNA-seq data illustrating changes in the β-catenin destruction complex (GO:0030877) geneset between control and JPI-547 treatment in HPAF-II. **(B)** Cycloheximide (50 μg/mL) chase assay with or without JPI-547 treatment followed by immunoblot analysis of AXIN2 levels. Representative images from three independent experiments are displayed (left), with relative AXIN2 levels quantified using ImageJ software (right) presented as the mean ± SD (n = 3); p-values by two-way ANOVA. **(C)** Immunoblot analysis of AXIN2 protein levels in HPAF-II cells following treatment with 1 μM JPI-547 for 0, 24, or 72 h. **(D)** Immunoblot analysis of β-catenin or non-phospho (active) β-catenin (Ser33/37/Thr41) levels in the nucleus after 72 h treatment with 2.5 µM olaparib or JPI-547. Lamin B1 was used as a nucleus loading control. **(E)** Upper: Representative images of active β-catenin immunohistochemical staining in AsPC-1 tumors collected from mice at the end of therapy. Lower: H-score calculated from n = 6 mice per group. Data represent mean ± SEM (n = 6); adjusted p-values by one-way ANOVA with Bonferroni's multiple comparisons test. **(F)** qRT-PCR analysis of c-Myc and AXIN2 after 120 h treatment with 2.5 µM olaparib or JPI-547. Data represent mean ± SD (n = 3); p-values by unpaired two-tailed Student's t-test. **(G)** Heatmap of RNA-seq data illustrating the expression levels of genes associated with Myc targets (HALLMARK_MYC_TARGETS_V1) between the indicated treatment groups in HPAF-II. **(H)** Gene Set Enrichment Analysis plot showing enrichment of the E2F targets geneset in untreated control samples compared to JPI-547-treated HPAF-II, based on transcriptomic data from RNA-seq. Normalized enrichment scores and nominal p-values were calculated using 1,000 permutations. **(I)** Bar graphs depicting the effect of CTNNB1 knockdown on the responsiveness of the indicated cell lines to JPI-547. Cells were transfected with the indicated small interfering RNAs for 24 h, exposed to JPI-547 for 120 h, and then subjected to MTT assays. Data represent mean ± SD (n = 3); p-values by two-way ANOVA. **(J)** Immunoblot analysis of β-catenin or non-phospho (active) β-catenin (Ser33/37/Thr41) expression in Capan-1 cells following Wnt ligand stimulation. Cells were stimulated with recombinant Wnt3a (50 ng/mL) and LiCl (10 mM) for 24 h, replenished with fresh culture medium, and then treated with 2.5 µM of olaparib and JPI-547 for 24 h before lysate preparation. Representative images from two independent experiments are shown.

We then focused on the impact of JPI-547 on the Wnt/β-catenin pathway. Analysis of transcriptomic data revealed notable upregulation of most genes within the β-catenin destruction complex (GO:0030877) upon JPI-547 treatment, except for AXIN2 (Figure 4A). This finding poses an intriguing discrepancy, considering the well-established role of TNKS1/2 in sustaining the Wnt pathway through degradation of AXIN1/2. As AXIN2 is a transcriptional target of β-catenin, we hypothesized that initial stabilization of AXIN2 by TNKS1/2 inhibition would suppress Wnt/β-catenin signaling. This suppression, in turn, would lead to feedback-mediated downregulation of AXIN2 transcription—resulting in a temporal mismatch between AXIN2 protein and mRNA levels, as previously observed with other TNKS inhibitors 51, 52. To explore this possibility, we performed cycloheximide chase assays and confirmed robust AXIN2 protein stabilization at 6 h post-treatment with JPI-547 in HPAF-II cells (Figure 4B). In contrast, no such stabilization was observed in Capan-1 cells, suggesting that this effect is context-dependent and may reflect underlying genetic alterations, such as RNF43 mutations. Furthermore, a time-course immunoblot analysis revealed a marked increase in AXIN2 protein levels at 24 h, followed by a decline at 72 h, although levels remained higher than in untreated controls (Figure 4C). This biphasic pattern suggests that AXIN2 is initially stabilized through TNKS inhibition, with the later decline in protein levels potentially reflecting transcript-level downregulation due to Wnt/β-catenin pathway suppression. To further support this mechanistic link, we next examined the subcellular distribution of β-catenin. In AsPC-1 cells, JPI-547 reduced nuclear translocation of active β-catenin (Figure 4D). JPI-547 also showed a trend toward reducing β-catenin activation in AsPC-1 tumors *in vivo*, although the effect was not significant (p = 0.0791), likely due to limited sample size (Figure 4E). qRT-PCR analyses confirmed that JPI-547 downregulated the expression of key target genes of the β-catenin pathway, Myc and AXIN2, specifically in PDAC cells with RNF43^mt^ (Figure 4F). In contrast, olaparib consistently induced Myc expression in all tested PDAC cell lines, reinforcing the direct interplay between TNKS1/2 and Wnt signaling (Figure 4G). In line with these findings, JPI-547 downregulated the mRNA levels of most of the downstream targets of Myc, and GSEA confirmed significant reduction in oncogenic E2F targets, whose expression depends in part on MYC activity (Figure 4H and I).

To determine the central role of β-catenin in mediating the susceptibility of PDAC cells with RNF43^mt^ to JPI-547, we depleted β-catenin using siRNA and found that its depletion neutralized the antiproliferative effect of JPI-547 in PDAC cells with RNF43^mt^ (Figure 4J). Moreover, JPI-547 significantly abrogated the expression of active β-catenin in Capan-1 cells under Wnt-stimulated conditions using rhWnt3a and LiCl treatment (Figure 4K).

In summary, these findings provide compelling mechanistic evidence that JPI-547 exerts its cytotoxic effects in PDAC cells with RNF43 mutations primarily through disruption of the Wnt/β-catenin signaling pathway.

### JPI-547 disrupts YAP pathway

**Figure 5:**
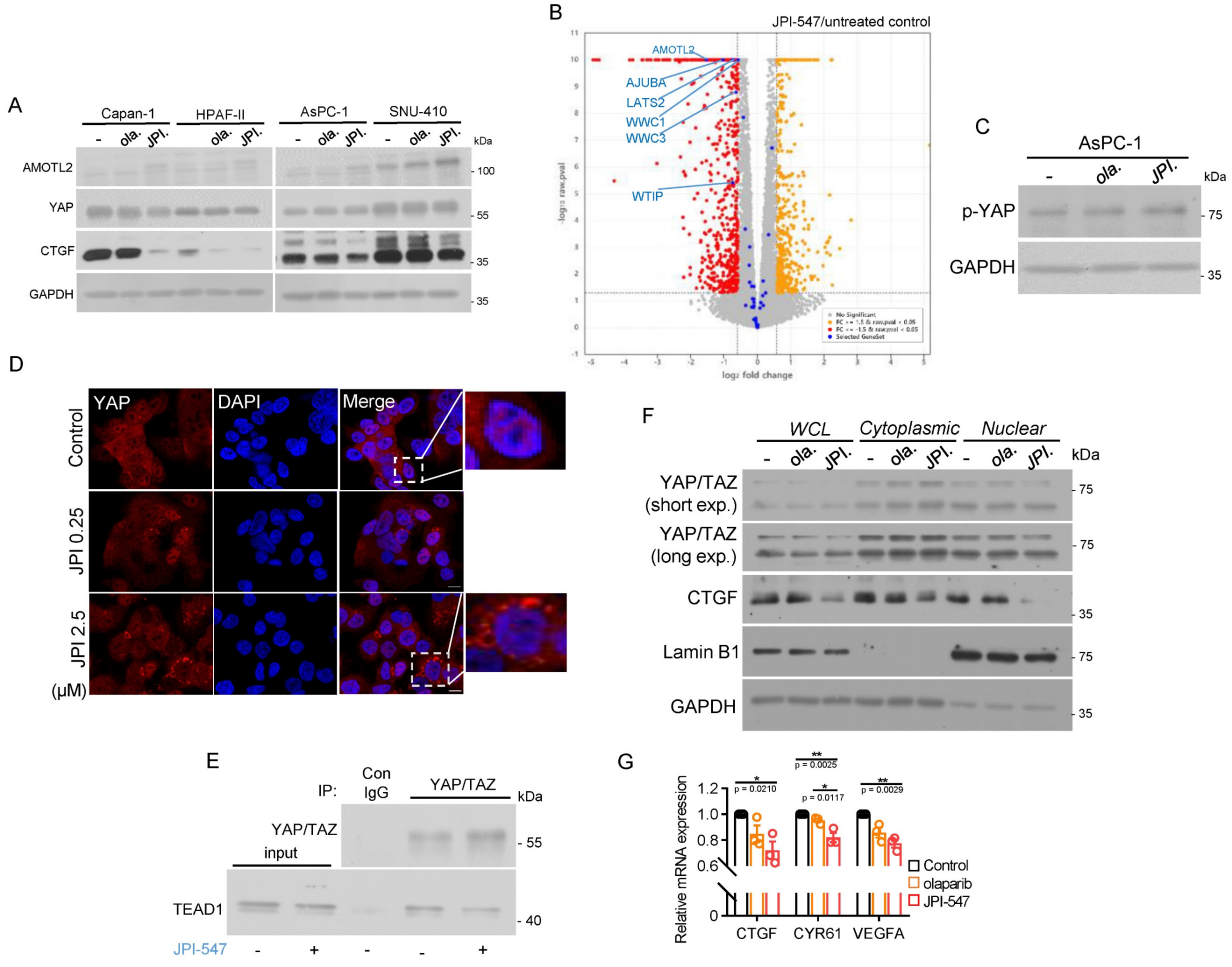
** JPI-547 disrupts YAP pathway. (A)** Immunoblot analysis of YAP-related proteins following treatment with 2.5 µM of olaparib or JPI-547 for 72 h in indicated cell lines. Representative images from duplicates are shown. **(B)** Volcano plot illustrating differentially expressed genes (DEGs) associated with the Hippo signaling pathway (GO:0035329) in HPAF-II cells. DEGs were identified from RNA-seq analysis of untreated and JPI-547-treated cells, defined by fold-change ≥ 1.5 and raw p < 0.05 (n = 3). **(C)** Immunoblot analysis of phospho-YAP (Ser127) following 72 h treatment with 2.5 µM olaparib or JPI-547 in AsPC-1. **(D)** Immunofluorescent imaging of YAP (red) in AsPC-1 cells treated with JPI-547 for 72 h. Representative images from three independent experiments are presented. **(E)** Immunoblot analysis demonstrating interactions between YAP/TAZ and TEAD1. AsPC-1 cells were treated with 2.5 µM JPI-547 for 72 h and subjected to immunoprecipitation. **(F)** Immunoblot analysis of YAP/TAZ and CTGF in subcellular fractions of AsPC-1 cells. Cells treated with 2.5 µM of olaparib or JPI-547 for 72 h underwent subcellular fractionation and subsequent immunoblotting. Lamin B1 served as a nuclear loading control. **(G)** qRT-PCR analysis of YAP transcriptional targets (CTGF, CYR61, and VEGFA) in AsPC-1 cells following 72 h treatment with 2.5 µM of olaparib or JPI-547. Data represent mean ± SEM (n = 3); adjusted p-values by one-way ANOVA with Bonferroni's multiple comparisons test.

TNKS has also been implicated in activation of the oncogenic YAP pathway, where the expression levels of its core components correlate with poor prognosis in PDAC 53, 54. This effect occurs through the promotion of RNF146-dependent ubiquitination and degradation of the angiomotin (AMOT) family of proteins, which act as negative endogenous regulators of oncogenic YAP 55. Hence, TNKS inhibitors hold promise for suppressing the pro-tumorigenic function of YAP signaling, which is frequently dysregulated in PDAC. JPI-547 consistently increased AMOTL2 protein levels, indicating its enhanced stability across all tested PDAC cell lines (Figure 5A). CTGF, a representative downstream target and surrogate marker of the YAP pathway, was significantly downregulated upon JPI-547 treatment, but not in response to olaparib (Figure 5A). These results suggest that YAP suppression is directly mediated by TNKS1/2 inhibition. Analysis of RNA sequencing data from Capan-1 cells revealed that JPI-547 downregulated genes in the Hippo pathway (GO:0035329) (Figure 5B). At the molecular level, JPI-547 induced phosphorylation of YAP at Ser127, indicating its inactive cytoplasmic retention (Figure 5C). This finding was substantiated by immunofluorescence analysis, which demonstrated the ability of JPI-547 to prevent nuclear YAP translocation, resulting in YAP retention in the cytoplasm. (Figure 5D). JPI-547 treatment disrupted the direct interaction between YAP/TAZ and TEAD1, which is an integral step in complete activation of YAP signaling (Figure 5E). Additionally, nuclear levels of YAP/TAZ were decreased following JPI-547 treatment in AsPC-1 cells, further indicating inhibition of the YAP pathway (Figure 5F). Finally, qRT-PCR confirmed reduced transcription of key YAP target genes (Figure 5G). Collectively, these results provide compelling evidence that JPI-547 inhibits the YAP pathway, with an agonistic effect regardless of the HRD status or Wnt addiction, improving the understanding of the mechanism of action of JPI-547.

## Discussion

We demonstrated that a novel dual inhibitor of PARP1/2 and TNKS1/2 suppressed the growth of PDAC cells, as characterized by HRD. Compared with existing PARPi, JPI-547 showed better antiproliferative effects, except for that of talazoparib, whose enhanced PARP-trapping ability has been described in relation to toxicity issues 56. JPI-547 showed an increased ability to suppress PARP catalytic activity while showing PARP trapping ability comparable to that of olaparib. The *in vivo* antitumor efficacy of JPI-547 against HRD-treated PDAC cells was slightly higher than that of olaparib; however, the difference was not significant. Thus, in the context of tumor growth suppression, the efficacy of JPI-547 in HRD cells was comparable to that of olaparib, as targeting PARP1/2 alone was sufficient to eliminate HR-deficient cancer cells. Furthermore, JPI-547 exhibited specific immune modulation in cancer cells with HRD, likely through activation of the cGAS-STING pathway, which is associated with increased tumor immunogenicity and potential responsiveness to immune checkpoint inhibitors. Additionally, IFN-I signaling was significantly upregulated by JPI-547, even in HPAF-II cells, suggesting that the immunostimulatory effect of JPI-547 extends beyond cells with HRD (Figure S4A). This effect was corroborated by the increased production of IFN-I and IFN-stimulated genes and secretion of CCL5 and CXCL10 in HPAF-II cells (Figure S4B, C). The pronounced potentiation of IFN signatures and major histocompatibility complex machinery in response to JPI-547 indicates potential for immunotherapeutic applications and calls for further translational research, including combination strategies with immune checkpoint inhibitors. While these findings suggest immunotherapeutic relevance, the implications should be interpreted with caution, as our study lacks validation in immunocompetent models. Future investigations in physiologically relevant immune-intact systems, such as syngeneic mouse models or humanized mice that recapitulate intact immune interactions, will be essential to fully evaluate the immune-modulatory effects of JPI-547 within a functional tumor microenvironment.

Although conducted in athymic mice lacking adaptive immunity, we observed elevated levels of circulating 2′3′-cGAMP following JPI-547 treatment in Capan-1 xenograft models. The DetectX® 2′3′-cGAMP assay used is species-independent and thus cannot differentiate whether the 2'3'-cGAMP originated from human tumor cells or host murine cells, posing a limitation in source attribution. Nevertheless, existing studies have primarily implicated cancer cells as the main source of extracellular 2'3'-cGAMP, whereas secretion by host immune cells remains less well characterized. Regardless of the precise origin, this finding provides *in vivo* evidence of cGAS-STING pathway engagement by JPI-547. As 2′3′-cGAMP is rapidly degraded by the ectonucleotidase ENPP1, its detectable accumulation in serum may suggest escape from ENPP1-mediated clearance or modulation of its enzymatic activity—possibilities not addressed in the present study 57. In addition, recent reports indicate that STING pathway activation can occur in tumor vasculature, raising the possibility that endothelial cells may also contribute to the observed increase 58. Further studies are needed to clarify these potential sources.

Although cells with germline *BRCA1/2* mutations are sensitive to PARP inhibition because of their inability to repair DNA double-strand breaks caused by replication fork collapse following prolonged PARP trapping, there remains a lack of consensus in clinical practice regarding the detection of HRD. Efforts to explore other HR genes such as *PALB2*, *ATM*, *CHEK2*, and *FANC* as predictive markers have not yielded definitive solutions. Some patients with deleterious BRCA1/2 variants do not respond to PARPi; in contrast, patients without mutations in core DNA damage repair genes show occasional clinical responses 59. Thus, there is strong interest in identifying and expanding the number of patients who can benefit from PARPi.

Our study extended beyond HRD and highlighted the potent antitumor effects of JPI-547 in PDAC cells with genetic Wnt addiction driven by pathogenic RNF43 variants. These effects are primarily mediated by suppression of the Wnt/β-catenin and Hippo YAP pathways, rather than by mimicking HRD function. Although JPI-547 treatment led to partial downregulation of HR-related gene sets in RNF43-mutant PDAC cells, this was not accompanied by measurable impairment in HR activity. This apparent disconnect may be attributed to multiple factors. First, post-transcriptional mechanisms such as protein stabilization or translational regulation may maintain effector protein levels despite modest transcript-level suppression. Second, HR repair depends on the localized recruitment of a limited pool of key proteins, and moderate reductions in total protein levels may not compromise repair competence. Such mechanistic explanations may help account for the observed disconnect, and are consistent with evolving clinical approaches in which transcriptomic data alone are not considered sufficient to define HRD status, thereby motivating integrated multi-omics strategies to improve the accuracy of HRD assessment 60, 61.

As described in our transcriptomic analysis, JPI-547 treatment in HPAF-II cells was associated with transcriptional upregulation of multiple β-catenin destruction complex components, including APC, GSK3B, and CSNK1A1—an effect that extends beyond the well-established AXIN2 protein stabilization mediated by TNKS inhibition (Figure 4A). This unexpected transcript-level increase is unlikely to reflect classical feedback regulation and may instead represent an adaptive transcriptional rewiring aimed at stabilizing the Wnt-inhibited state. Beyond its catalytic PARP activity, TNKS functions as a multivalent scaffold critical for the structural integrity of β-catenin degradasome 62. Its inhibition could possibly disrupt protein-protein interaction networks, indirectly altering the localization or activity of transcriptional regulators. These structural perturbations may lead to secondary transcriptional responses, though the precise mechanisms remain to be elucidated.

Although our investigation focused on RNF43 in PDAC, our findings suggest the applicability of JPI-547 to other tumor types with RNF43 mutations. In addition, studies are needed to determine the efficacy of JPI-547 in various cancers harboring different genetic aberrations, such as R-spondin fusions and APC mutations, that confer Wnt pathway dependency. A promising application of JPI-547 is in cancers characterized by mutations in the tumor suppressor APC, which is frequently observed during early tumorigenesis, including in colorectal cancer 63. These genetic alterations render APC-mutated tumors attractive candidates for JPI-547, potentially broadening the eligible patient population. Preclinical data supporting the antitumor effects of TNKS inhibitors against APC mutation-driven cancers provide a strong foundation for pursuing this therapeutic strategy 52, 64. In support of this, COLO-320DM (APC^p.S811*) and DLD-1 (RNF43^X659fs and RNF43^L214M), two colorectal cancer cell lines harboring Wnt pathway alterations, showed greater sensitivity to JPI-547 than to olaparib (Figure S5). While preliminary and limited to cytotoxicity assessment, these findings support further evaluation of JPI-547 in Wnt-addicted colorectal cancers. Moreover, previous studies have identified somatic mutations in APC in a subset of PDAC cases, suggesting that a considerable number of patients with PDAC also exhibits Wnt pathway addiction 65, 66. The positive correlation between JPI-547 sensitivity and basal β-catenin expression levels suggests the utility of tumor immunohistochemistry scores for β-catenin as a stratification marker beyond genetic alterations in the selection of patients with PDAC.

In conclusion, our study highlights the potent antitumor activity of JPI-547 in PDAC with HRD, highlighting its potential as a therapeutic agent in patients with PDAC. By concurrently targeting TNKS1/2, JPI-547 introduced a therapeutic strategy for patients with Wnt-driven PDAC to address unmet medical needs in this challenging clinical context. These findings may advance drug development and improve the outcomes of patients with PDAC and other malignancies characterized by similar genetic alterations.

## Supplementary Material

Supplementary figures.

## Figures and Tables

**Table 1:** IC_50_ values of various PARPi against Capan-1 cells in MTT assays.

Compound	IC_50_ values (µM)
JPI-547	0.1895
olaparib	1.105
talazoparib	0.02045
niraparib	0.5239
veliparib	>10
rucaparib	1.55

IC50 values marked as '>10' indicate concentrations where 50% inhibition was not achieved. IC50 values were calculated using Prism GraphPad 8.0.1 with normalized response data.

## Abbreviations

PDAC: pancreatic ductal adenocarcinoma
PARP: poly(ADP-ribose) polymerase
HR: homologous recombination
HRR: homologous recombination repair
HRD: homologous recombination deficiency
TNKS: tankyrase
PAR: poly(ADP) ribose
Wnt: wingless-related integration site
RNF43: ring finger protein 43
LRP6: low-density lipoprotein receptor-related protein 6
RNF146: ring finger protein 146
BRCA1/2: breast cancer gene 1/2
YAP1: yes-associated protein 1
CYR61: cysteine-rich angiogenic inducer 61
VEGFA: vascular endothelial growth factor A
MYBL2: MYB proto-oncogene-like 2
GSEA: gene set enrichment analysis
AMOTL2: angiomotin-like protein 2
CTGF: connective tissue growth factor
