# Supplementary material for: JPI-547, a Dual Inhibitor of PARP/Tankyrase, Shows Antitumor Activity Against Pancreatic Cancers with Homologous Recombination Repair Deficiency or Wnt-Addiction
Source: Int J Biol Sci. 2025 Aug 22;21(12):5460–75. doi: 10.7150/ijbs.113726 (PMC12435476; doi:10.7150/ijbs.113726)
Supplement: Supplementary file 1 — Supplementary figures. [file ijbsv21p5460s1.pdf]

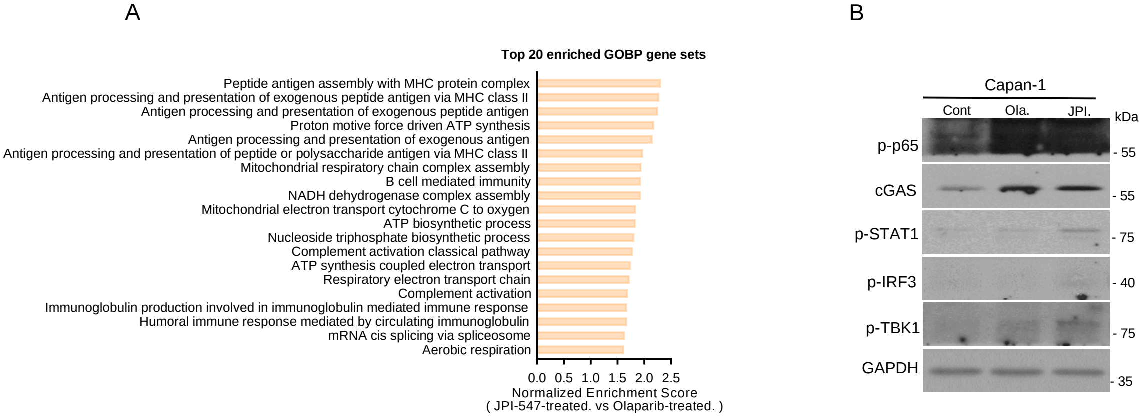

Figure S1. Cancer cell-intrinsic immunostimulatory effect of JPI-547. (A) The top 20 enriched GOBP gene sets in Capan-1 cells after treatment with JPI-547 compared to olaparib, ranked by normalized enrichment score from GSEA. (B) Immunoblot analysis of the cGAS-STING pathway in Capan-1 cells following treatment with 2.5 uM of olaparib or JPI-547 for 120 h. (C) Immunofluorescence analysis of cGAS (red) and p-TBK1 (Ser172) (green) in Capan-1 cells after treatment with 2.5 uM of olaparib or JPI-547 for 72 h. (D) The top 20 enriched GOBP gene sets in HPAF-II cells following JPI-547 treatment compared to olaparib treatment, ranked by normalized enrichment score from GSEA.

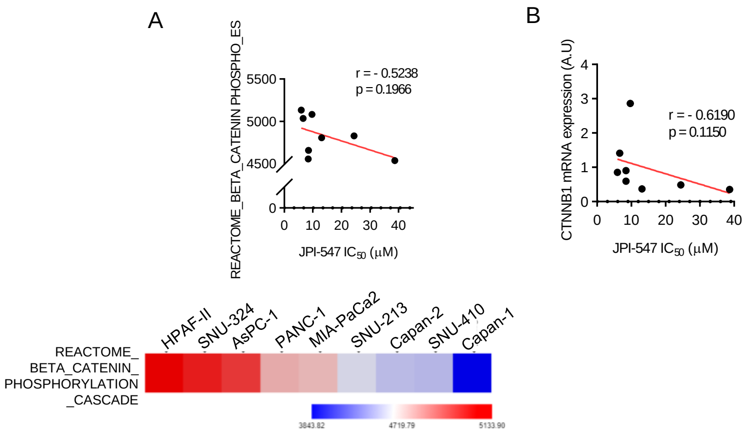

Figure S2. Transcriptomic analysis of the β-catenin pathway and β-catenin mRNA expression did not exhibit a significant correlation with JPI-547 sensitivity in PDAC cell lines. (A) β-catenin pathway dependency scores were calculated using single-sample GSEA based on transcriptomic data from the CCLE database (DepMap Public 22Q2). Correlation analysis was performed using nonparametric Spearman correlation (two-tailed). (B) Correlation analysis using normalized β-catenin mRNA expression levels extracted from the CCLE database was conducted using nonparametric Spearman correlation (two-tailed).

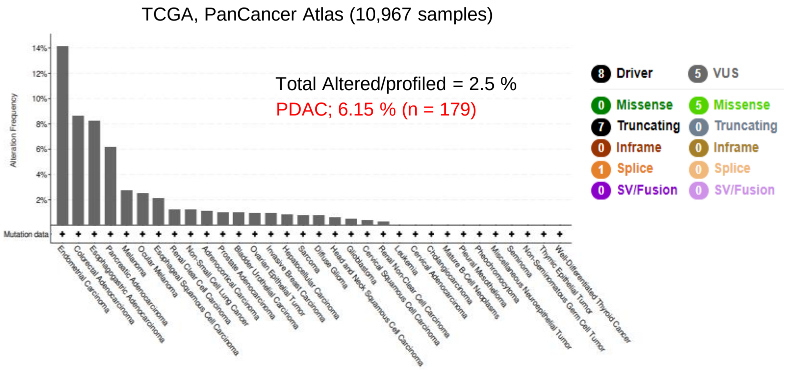

Figure S3. The frequencies of RNF43 alterations across solid tumors. Bar graph was plotted in cBioPortal using TCGA, PanCancer Atlas dataset.

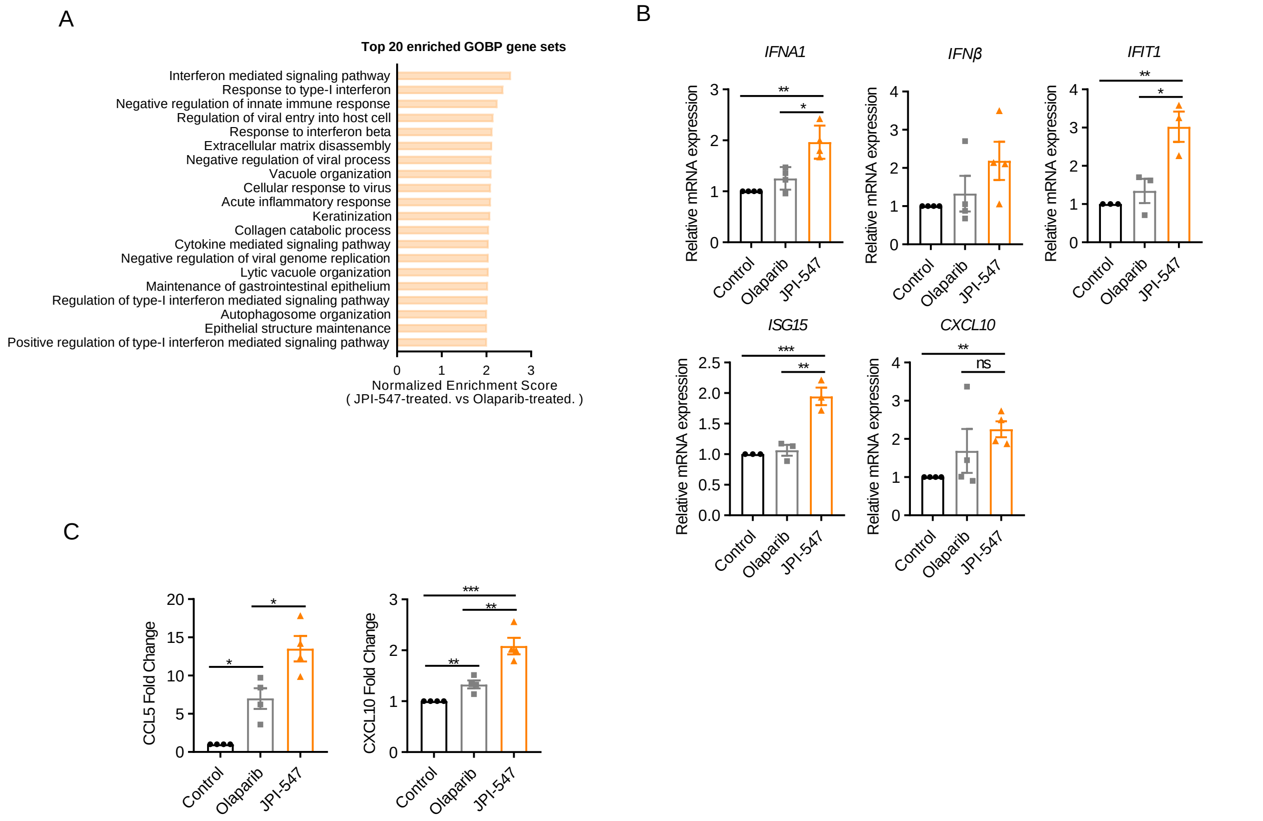

Figure S4. JPI-547 induces proinflammatory factors in HPAF-II. (A) The top 20 enriched Gene Ontology Biological Process (GOBP) gene sets in HPAF-II cells following JPI-547 treatment compared to olaparib, ranked by normalized enrichment score from GSEA. (B) qRT-PCR analysis of IFN-I and interferon-stimulated genes after 72 h treatment with 1 μM olaparib or JPI-547. Data represent mean ± SEM ($n \geq 3$); adjusted p-values by one-way ANOVA with Bonferroni's multiple comparisons test, ns, not significant, * $p < 0.05$, ** $p < 0.005$, *** $p < 0.001$. (C) ELISA quantification of secreted CCL5 and CXCL10 after 72 h treatment with 1 μM olaparib or JPI-547. Data represent mean ± SEM ($n = 4$); adjusted p-values by one-way ANOVA with Bonferroni's multiple comparisons test, * $p < 0.05$, ** $p < 0.005$, *** $p < 0.001$.

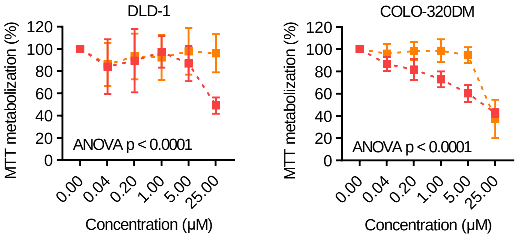

Figure S5. Wnt-addicted colorectal cancer cell lines exhibit increased sensitivity to JPI-547 compared to olaparib. Dose–response curves illustrating the cytotoxic effects of JPI-547 and olaparib in DLD-1 and COLO-320DM cells.Data represent mean ± SEM (n = 3); p-values by two-way ANOVA.
